# Loss of integrin alpha7-mediated signaling induces a dendritic cell-like phenotype in macrophages cultured on laminin-211/221 isoforms

**DOI:** 10.1016/j.jbc.2025.110419

**Published:** 2025-06-25

**Authors:** Nagako Yoshiba, Tomoki Maekawa, Kiyotoshi Sekiguchi, Masaru Kaku, Kridtapat Sirisereephap, Meircurius Surboyo, Yurie Sato-Yamada, Andrea Rosenkranz, Akihiro Hosoya, Naoto Ohkura, Yoshito Kakihara, Takeyasu Maeda, George Hajishengallis, Kenji Izumi, Kunihiko Yoshiba

**Affiliations:** 1 Division of Oral Science for Health Promotion, Department of Oral Health and Welfare, Graduate School of Medical and Dental Sciences, Niigata University, Niigata, Japan; 2 Center for Advanced Oral Science, Graduate School of Medical and Dental Sciences, Niigata University, Niigata, Japan; 3 Division of Matrixome Research and Application, Institute for Protein Research, Osaka University, Suita, Osaka, Japan; 4 Division of Bio-Prosthodontics, Department of Oral Health Science, Graduate School of Medical and Dental Sciences, Niigata University, Niigata, Japan; 5 Division of Histology, School of Dentistry, Health Sciences University of Hokkaido, Ishikari-Tobetsu, Hokkaido, Japan; 6 Division of Cariology, Operative Dentistry and Endodontics, Department of Oral Health Science, Graduate School of Medical and Dental Sciences, Niigata University, Niigata, Japan; 7 Division of Dental Pharmacology, Department of Oral Health Science, Graduate School of Medical and Dental Sciences, Niigata University, Niigata, Japan; 8 Department of Basic and Translational Sciences, Laboratory of Innate Immunity and Inflammation, School of Dental Medicine, University of Pennsylvania, Philadelphia, Pennsylvania, USA; 9 Division of Biomimetics, Department of Oral Health Science, Graduate School of Medical and Dental Sciences, Niigata University, Niigata, Japan

**Keywords:** laminin, integrin, macrophage, dendritic cell, phosphatidylinositide 3-kinase (PI 3-kinase), Akt PKB, GM-CSF

## Abstract

Laminin comprises α/β/γ subunits and performs tissue-specific functions that control cellular behavior. Laminin-α2 chains are highly expressed in neural components such as glial and Schwann cells and in muscles. Macrophages play important roles in tissue homeostasis and repair, and laminins affect macrophage dynamics. Integrin α7, a transmembrane receptor crucial for regulating cell–matrix interactions, has a high affinity for laminin-α2, but its function in macrophages remains unknown. Here, we find that loss of integrin α7 signaling induces a dendritic cell (DC)-like phenotype in THP-1-derived macrophages and in primary monocytes-derived macrophages induced by granulocyte macrophage colony-stimulating factor cultured on laminin-α2 chains. Functional blocking of integrin α7 induced dendritic processes of THP-1-derived macrophages. Gene expression analysis revealed DC markers and costimulatory molecules, and coculture experiments demonstrated that the DC-like cells could stimulate T cell proliferation. Functional inhibition of integrin α7 decreased PI3K-p85α levels and activated PI3K, thereby activating AKT. Monocyte-derived macrophages cultured on laminin α2 chains decreased integrin α7 expression, exhibited dendritic-like morphology, and increased expression of DC markers and costimulatory molecules. These findings suggest that, besides the established influence of cytokine milieu, DC differentiation is regulated by laminin α2/integrin α7-mediated cell adhesion. Integrin α7 has been a therapeutic target in tumors, and antibody-based integrin α7 neutralization can be clinically useful. The results of this study suggest implications for integrin α7 and laminin-α2 chains in DC immunotherapy.

The basement membrane is a sheet of highly specialized extracellular matrix that surrounds muscle and fat as well as axons and covers the basal surface of epithelial and endothelial cells (1). Laminin is a major basement membrane protein composed of α, β, and γ chains. Laminins regulate cell adhesion, migration, survival, and differentiation, as well as maintenance of phenotype (2). Five α- (α1–5), three β- (β1–3), and three γ- (γ1–3) laminin chains are known, which assemble into at least 16 isoforms *in vivo*. Chain composition reflects isoform nomenclature: *e*.*g*., α2β1γ1 laminin is called laminin-211. These isoforms have tissue- and developmental stage-specific distribution patterns—laminin-511 is a ubiquitous basement membrane component, whereas laminins containing α2 are more cell-type specific (2). Laminin-211 is highly expressed in Schwann cells in peripheral nerves (3, 4), and oligodendrocytes and astrocytes in the central nervous system (5) as well as in thymic epithelium (6) and skeletal muscle (7). Laminin-221 is most prominent in the basement membrane of cardiac muscle (8).

Macrophages are highly plastic and heterogeneous immune cells that play an essential role in wound healing, host defense, and the tumor microenvironment (9, 10). Macrophages exhibit a phenotypic transition that is classified into two opposing types: the classical (proinflammatory: M1) and the alternative (anti-inflammatory and prohealing: M2), which in fact represent the extremes of a continuum of activation states (11, 12). Macrophages with the CD163^+^ M2 phenotype are closely associated with Schwann cells (4, 13). Laminin-211, an isoform of laminin expressed on the surface of Schwann cells, regulates the expression of CD163 (4), a neuroprotective subset of macrophages (14).

Macrophages express integrins on their cell surface (10). Integrins are heterodimeric transmembrane matrix receptors composed of α and β subunits that exhibit distinct ligand-binding properties and mediate cell–cell or cell–extracellular matrix interactions. There are 24 types of integrins in mammals, of which four (α3β1, α6β1, α6β4, and α7β1) bind primarily to laminin, and their binding affinities are isoform-specific (15). Laminin-α2 exhibits a high affinity for integrin α7β1 but demonstrates poor affinities for integrins α3β1, α6β1, and α6β4. By contrast, laminin-α5 has high affinity for integrins α3β1, α6β1, and α6β4 (15). The interaction between laminin and integrins activates downstream signaling pathways (16).

Laminins affect the immune system (17). Endothelial laminin-511 promoted monocyte-to-macrophage differentiation upon change in integrin expression (18). However, the details of laminin’s action on immune cells and the molecular pathways involved are largely unknown (17). We demonstrated that all laminin-associated integrin α3/α6/α7 mRNAs are expressed in THP-1-derived macrophages cultured on laminin-211/511 (4). Integrin α7, in particular, has a high affinity for laminin containing α2 chains, and its expression level is significantly increased in THP-1 macrophages cultured on laminin-211 (4). However, the function of integrin α7 in macrophages is unknown (17).

Here, we find that loss of integrin α7 signaling induces a dendritic cell (DC)-like phenotype in macrophages cultured on laminin-α2-coated plates. Integrin α7 and laminin-α2 are aberrantly expressed in glioblastoma, the most lethal primary brain tumor, and inhibiting integrin α7 is expected to have therapeutic effects (19, 20, 21, 22, 23). DC-based cancer immuno-cell therapy has exhibited remarkable clinical benefits (24). Thus, the results of the present study have implications for clinical applications targeting integrin α7 and laminin-α2 and for DC-based immuno-cell therapy.

## Results

### Localization of laminin-associated integrins is differentially regulated in the presence of laminin isoforms

**Figure 1:**
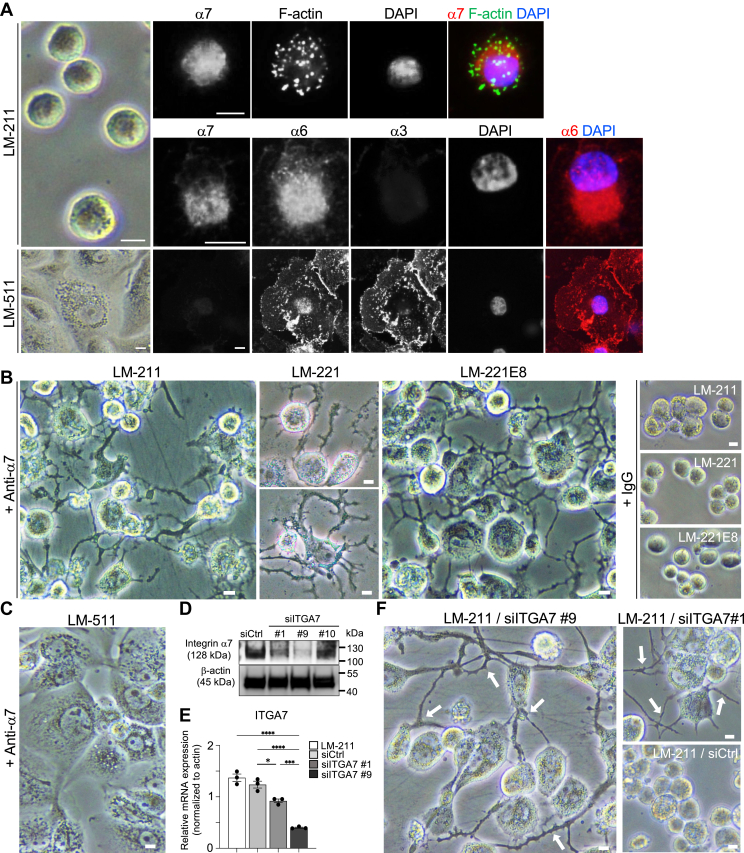
**Localization of laminin-related integrins and the effect of functional blocking of integrin α7 in THP-1 macrophages cultured on laminin-211/221/221E8 or laminin-511 for 5 days**. *A*, morphology and multiple immunofluorescent staining for laminin-related integrins (α7, α6, and α3) and phalloidin in THP-1 macrophages cultured on laminin (LM)-211/-511. Nuclei were counterstained with DAPI. Immunostaining was performed four times independently, imaging a total of 300 to 350 cells per antibody. *B*, morphology of THP-1 macrophages cultured on laminin-211/-221/-221E8 in the presence of integrin α7 function-blocking antibodies (anti-α7) or isotype control IgG. In the presence of integrin α7 function-blocking antibodies, dendritic-like branching and extended cellular processes are observed. These experiments were repeated at least five times independently. *C*, morphology of THP-1 macrophages cultured on laminin-511 in the presence of integrin α7 function-blocking antibodies. *D*, western blotting of integrin α7 under conditions of integrin α7-targeted small interfering RNA (siITGA7) on day 5. siITGA7 #9 and #1 reduced integrin α7 levels on laminin-211. Nonspecific siRNA was used as control (siCtrl); β-actin was used as a loading control. *E*, gene expression levels of integrin α7. Data are presented as the mean ± SEM of three independent experiments. ∗*p* < 0.05, ∗∗∗*p* < 0.001 and ∗∗∗∗*p* < 0.0001 as determined by Tukey’s *post hoc* test. *F*, morphology of THP-1 macrophages treated with integrin α7-targeted small interfering RNA #9, #1 or nonspecific siRNA on day 5. The knockdown experiment was repeated three times independently. Integrin α7 knockdown induced dendritic processes in THP-1 macrophages cultured on laminin-211 (*arrows*). Nonspecific siRNA did not induce obvious morphological alterations. The *scale bars* represent 10 μm. DAPI, 4′,6-diamidino-2-phenylindole; IgG, immunoglobulin G.

Because little is known about the localization pattern of laminin-associated integrins in macrophages, we evaluated protein expression using anti-integrin α7/6/3 antibodies in THP-1 macrophages cultured on laminin-211- or −511-coated dishes. THP-1 macrophages cultured on laminin-211-coated plates were spherical (Fig. 1*A*). We found F-actin-rich puncta called podosomes, which form close contacts with the extracellular matrix and are constitutively detected in the myeloid lineage (macrophages, osteoclasts, and DCs) (25), in THP-1 macrophages. Merged images of F-actin and integrin α7 showed partial localization of integrin α7 in cells on day 5 of culture (Fig. 1*A*). Unexpectedly, we could not find integrin α3 staining in THP-1 macrophages cultured on laminin-211 (Fig. 1*A*). Integrin α6 was more diffuse than integrin α7 (Fig. 1*A*). By contrast, macrophages cultured on laminin-511 were expanded in shape and devoid of integrin α7 localization (Fig. 1*A*). Integrins α3 and α6 were localized throughout the cell on laminin-511 (Fig. 1*A*). These data are consistent with our previous results showing that THP-1 macrophages cultured on laminin-211 or laminin--511 have higher mRNA expression of integrin α7 or integrins α3 and α6, respectively (4).

### Functional blocking of integrin α7 on laminins containing the α2 chain induces dendrite formation in THP-1 derived macrophages

Based on the prominent staining of integrin α7 on laminin-211 (Fig. 1*A*), we evaluated the effect of integrin α7 on laminin-211 using an antibody that blocks integrin α7 function. Surprisingly, on laminin-211-coated plates, branching and extended cellular processes similar to dendrites were seen (Fig. 1*B*). Because the E8 domains of the globular domains in laminin α chains are prerequisites for integrin-binding to laminins (2, 16), we cultured THP-1 macrophages on another isoform of an α2-chain-containing laminin, laminin-221, and on the recombinant E8 fragments of laminin-221 (laminin-221E8), which are the minimum fragments that confer integrin-binding activity (26, 27). In the presence of the integrin α7 antibody, extended dendrites were seen on laminin-221 and laminin-221E8 (Fig. 1*B*). By contrast, the same dose of an isotype control immunoglobulin G (IgG) did not affect the morphology on laminin-211/221/221E8 (Fig. 1*B*). Upon treatment with anti-integrin α7 blocking antibody, THP-1 macrophages did not produce dendrites when cultured in the presence of laminin-511 (Fig. 1*C*), confirming the low affinity of integrin α7 for laminin-511. We next explored the effect of siRNA knockdown of integrin α7 in THP-1 macrophages cultured on laminin-211. Integrin α7 siRNA #9 as well as #1 reduced protein (Fig. 1*D*) and mRNA (Fig. 1*E*) expression. siRNA-mediated knockdown of integrin α7 resulted in the formation of extended dendrites from THP-1 macrophages (Fig. 1*F*). These morphological changes induced by functional blockade or siRNA knockdown of integrin α7 highlight the effect of integrin α7 on the production of dendrite-like structures from THP-1 macrophages cultured with laminin α2 chains.

### Suppression of integrin α7 expression upregulates antigen binding and co-stimulating molecules, leading to DC-like cell differentiation

**Figure 2:**
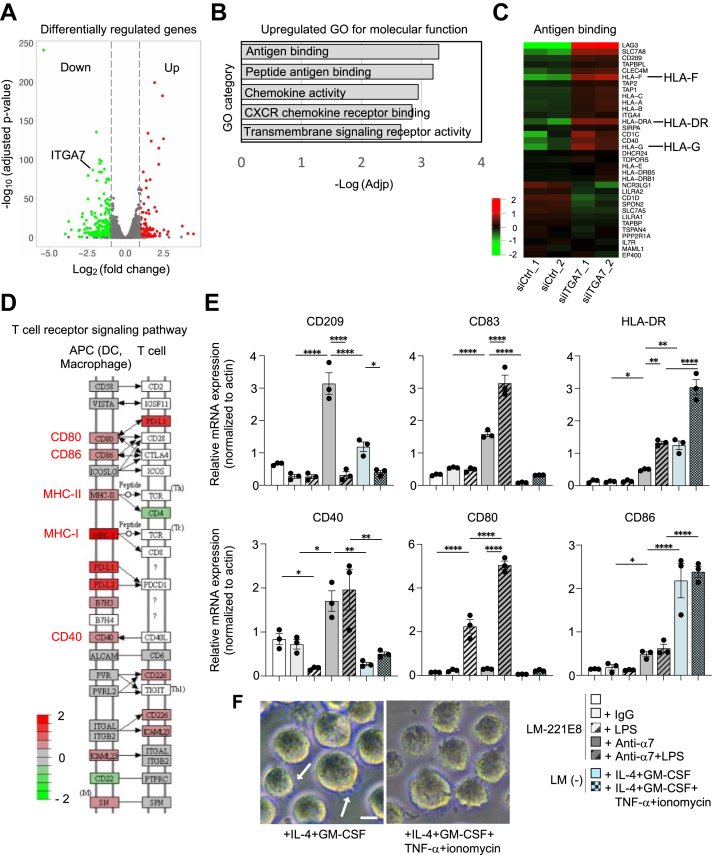
**Dendritic cell markers are upregulated by siRNA-mediated downregulation or functional blocking of integrin α7 in THP-1 macrophages cultured on laminin-211 or 221E8 for 5 days**. *A*, volcano plot illustrating differentially regulated gene expression from RNA-Seq analysis in THP-1 macrophages transfected with integrin α7 siRNA compared with nonspecific siRNA. *B*, gene ontology (GO) enrichment analysis of genes upregulated for molecular function. The top five most significantly affected categories are shown. *C*, heatmap depicting the expression of genes in the antigen-binding signature. Integrin α7-targeted small interfering RNA (siITGA7) transfected cells compared with the nonspecific control siRNA (siCtrl) (n = 2 for each group). *D*, KEGG pathway analysis of cell adhesion molecules between antigen-presenting cells (APCs) and T cell receptor signaling. *E*, analysis of gene expression in THP-1 macrophages cultured under the conditions indicated. Data are presented as the mean ± SEM of three independent experiments. ∗*p* < 0.05, ∗∗*p* < 0.01, and ∗∗∗∗*p* < 0.0001 as determined by Tukey’s *post hoc* test. LM-221E8: laminin-221E8; Anti-α7: anti-integrin α7 antibody. *F*, morphology of THP-1 macrophages treated with the conditions indicated. *Arrows* represent spike-like projections. The *scale bar* represents 10 μm. KEGG, Kyoto Encyclopedia of Genes and Genomes.

Gene expression in THP-1 macrophages transfected with siRNA #9 targeting integrin α7 was analyzed using RNA sequencing (RNA-Seq) followed by process enrichment and pathway analyses. We found 113 significantly upregulated genes and 316 downregulated genes, including integrin α7 as a downregulated gene (Fig. 2*A*). Gene ontology enrichment analysis showed that among the genes whose expression was elevated with respect to molecular function, the top-ranked significantly affected term was antigen binding (Fig. 2*B*), including human leukocyte antigen A-F (HLA-F), HLA-G, and HLA-DR (Fig. 2*C*). THP-1 macrophages functionally blocked integrin α7 and cultured in the presence of laminin α2 chains exhibited dendrite formation and upregulation of genes for antigen-binding molecules, suggesting that the macrophages differentiated into DC-like cells (28). DCs are the most potent antigen-presenting cells that stimulate T cells to proliferate by costimulatory molecules, such as CD40, CD80, and CD86 (28). We analyzed the genes regulating the T cell receptor signaling pathway using the Kyoto Encyclopedia of Genes and Genomes pathway analysis and found upregulation of these costimulatory molecules, in addition to antigen-binding genes MHC classes I and II (Fig. 2*D*), suggesting their ability to activate T cells.

### Lipopolysaccharide (LPS) upregulates antigen-binding and costimulatory molecules of THP-1 macrophages treated with integrin α7 function-blocking antibodies and cultured on laminin-221E8

Following antigen uptake, immature DCs convert to a mature phenotype, characterized by the upregulation of antigen-binding and costimulatory molecules (28). Because bacterial LPS are thought to induce maturation of immature DCs (28), we applied LPS and examined phenotypic changes in THP-1 macrophages treated with the anti-integrin α7 antibody and cultured on laminin-221E8. Expression of DC markers, CD209 (DC-SIGN), HLA-DR (MHC class II), and the regulator CD83 was higher with integrin α7 function-blocking antibodies compared with its absence (Fig. 2*E*). Activation by LPS further increased the expression of CD83 and HLA-DR, but decreased the expression of CD209 (Fig. 2*E*), consistent with previous observations (29, 30). Because CD209, which is abundantly expressed in immature DCs, is required for receptor-mediated endocytosis (31), the downregulation of CD209 by LPS suggests decreased endocytosis similar to the markedly decreased endocytic activity in mature DCs (32). The response of THP-1 macrophages to LPS was different in CD40 and CD80, with CD40 being downregulated and CD80 upregulated (Fig. 2*E*). LPS induced CD40 (33) and CD80 (34) gene expression in macrophages, but interleukin-10 (IL-10) inhibited LPS-induced CD40 expression (35). α2-containing laminins induce IL-10 expression in macrophages (4), which explains the attenuation of CD40 expression in LPS-stimulated macrophages.

The THP-1 cells treated with integrin α7 antibodies were compared to THP-1 cells treated with GM-CSF/IL-4 or GM-CSF/IL-4/TNF-α/ionomycin, a known model for immature and mature DCs, respectively (29). Expression of DC markers, CD209 and CD83, and costimulatory molecules, CD40 and CD80, was significantly higher in THP-1 macrophages treated with integrin α7 function-inhibitory antibodies, as compared to the established model induced by cytokines, *i*.*e*., DCs generated from THP-1 cells in the presence of appropriate cytokines (see Experimental procedures) (Fig. 2*E*). Furthermore, in the cytokine-induced model, CD209 expression was also decreased in mature DCs compared to immature DCs. Taken together, these data suggest that THP-1 macrophages cultured with laminin α2 chains in the presence of anti-integrin α7 antibody acquired a DC-like phenotype. As for the morphology, cytokine-treated THP-1 cells were different from those induced by anti-integrin α7 antibody, showing spike-like projections (Fig. 2*F*) as described (29).

### Morphological evidence of endocytosis by THP-1 macrophages treated with integrin α7 function-blocking antibodies and cultured on laminin-221E8

**Figure 3:**
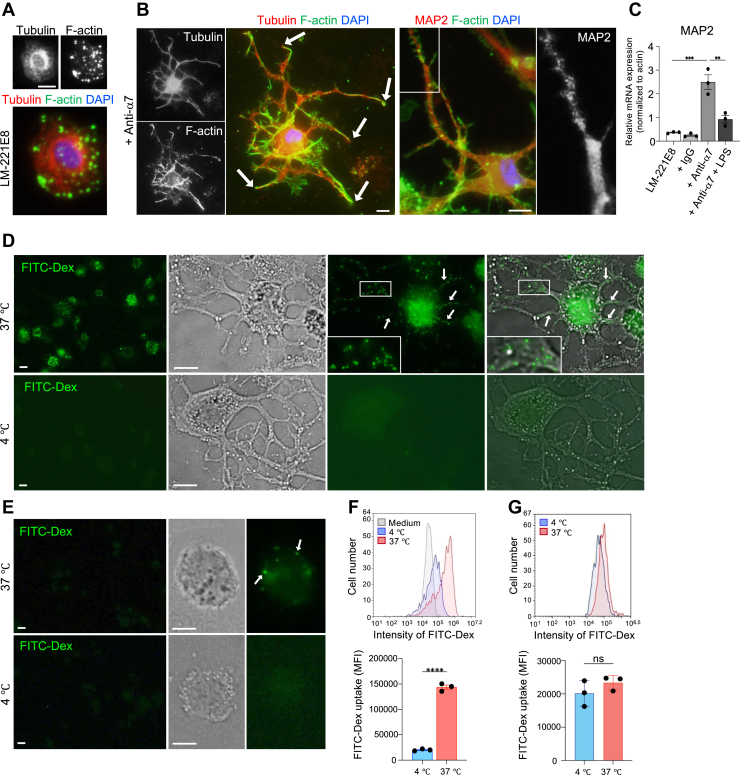
**Morphologic evidence of endocytosis by THP-1 macrophages treated with integrin α7 function-blocking antibodies on laminin-221E8**. *A*, immunofluorescent staining for phalloidin and microtubule in THP-1-derived macrophages cultured without integrin α7 function-blocking antibodies for 5 days. *B*, immunofluorescent staining for phalloidin, microtubule, and microtubule-associated protein 2 (MAP2) in THP-1-derived macrophages cultured with integrin α7 function-blocking antibodies (anti-α7) for 5 days. *Arrows* represent the tips of dendritic processes. An enlarged view of the MAP2 *box area* is shown on the *right*. *C*, analysis of gene expression of MAP2 in THP-1 macrophages cultured under the conditions indicated. Data are presented as the mean ± SEM of three independent experiments. ∗∗*p* < 0.01 and ∗∗∗*p* < 0.001 as determined by Tukey’s *post hoc* test. *D* and *E*, morphologic evidence for endocytosis. THP-1 macrophages treated with integrin α7 function-blocking antibodies (*D*) or not treated (*E*) were incubated with FITC-dextran at 37 °C or on ice (background control). *Arrows* indicate the same point on the immunofluorescence image or immunofluorescence + phase contrast image (*D*). The *boxed* area is shown in the inset. A large number of FITC^+^ puncta were evident in dendritic processes (*D*). Original magnification: × 200 (left lane) and × 600. *F* and *G*, FITC-dextran uptake of THP-1 macrophages treated with integrin α7 (*F*) or not treated (*G*) were analyzed by flow cytometry and statistical analysis. Data are representative of three independent experiments. Each column represents the mean ± SEM of three independent experiments. ns, not significant. ∗∗∗∗*p* < 0.0001 as determined by an unpaired *t* test. FITC-Dex: FITC-dextran. The *scale bars* represent 10 μm.

Immature DCs efficiently internalize antigens, in which endosome trafficking relies on the actin and microtubule cytoskeleton (36) as well as microtubule-associated proteins (MAPs) (37). The RNA-Seq data in the present study indicated high expression of intrinsic genes in integrin α7 siRNA-transfected macrophages compared with nonspecific siRNA-transfected control macrophages, including tubulin-encoding genes *TUBA4A* (103.59, 99.98/52.24, and 55.24), *TUBA1C* (436.5, 445/285.34, and 287.338), and *TUBB6* (198.74, 208.44/132.9, and 132.67), as well as the gene encoding *MAP2* (18.22, 12.42/4.61, and 6.36). Therefore, we investigated the localization of these proteins. Immunofluorescence showed that tubulin was densely distributed in perinuclear areas in macrophages without integrin α7 function-blocking antibodies (Fig. 3*A*). By contrast, F-actin and tubulin or MAP2 were present up to the tip of dendrites in DC-like cells (Fig. 3*B*). Because MAP2 expression is primarily reported in neuronal dendrites (38), *MAP2* expression was examined in integrin α7 function-blocking antibody-treated macrophages. *MAP2* expression increased by almost 5-fold compared with the no antibody control or IgG control and decreased by >50% after LPS stimulation (Fig. 3*C*). Next, we assessed the endocytic capacity of DC-like cells using FITC-dextran (32). A large number of FITC^+^ spots (0.5–3 μm in diameter) corresponding to FITC-dextran incorporating endosomes were detected in dendritic processes and cell bodies treated with integrin α7 function-blocking antibodies at 37 °C, whereas almost no spots were observed in control cells (4 °C) (Fig. 3*D*). In contrast, THP-1 macrophages not treated with anti-integrin α7 took up relatively little FITC-dextran (Fig. 3*E*). Histogram and statistical analysis showed significant levels of FITC-dextran uptake in THP-1 macrophages treated with integrin α7 function inhibitory antibody (Fig. 3*F*). In contrast, no significant differences were observed for THP-1 macrophages not treated with integrin α7 function inhibitory antibody (Fig. 3*G*).

### Integrin α7 function-blocking antibodies induce CD4^+^ and CD8^+^ T cell proliferation when cocultured with DC-like cells in the presence of laminin-221E8

**Figure 4:**
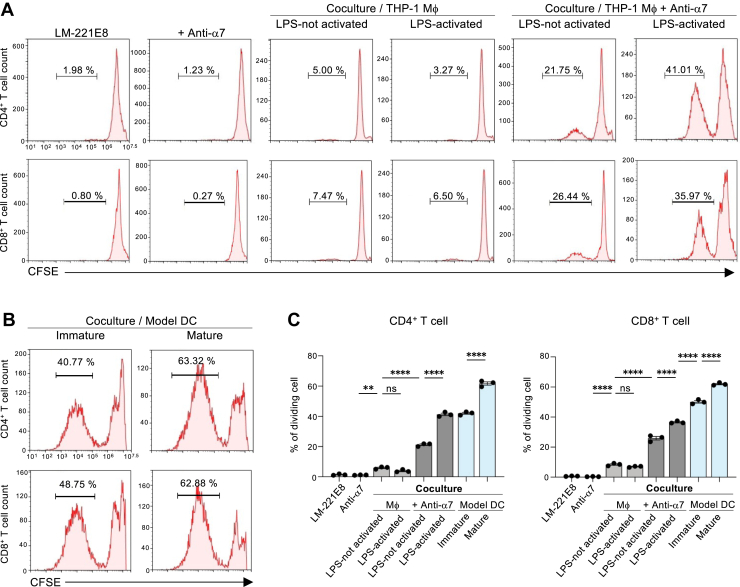
**Proliferation of CD4^+^ and CD8^+^ T cells after coculture with DC-like cells induced by integrin α7 function-blocking antibodies on laminin-221E8-coated plates.** CD4^+^ and CD8^+^ T cells were stained with CFSE before coculture. *A*, DC-like cells or THP-1 macrophages were stimulated with LPS (1 μg/ml) or left unstimulated for 24 h before coculture. Representative histograms showing the proliferation of CD4^+^ and CD8^+^ T cells under the indicated conditions are presented. *B*, THP-1 cells were treated with rhGM-CSF and rhIL-4 to generate immature model DCs, or with rhGM-CSF, rhIL-4, rhTNF-α, and ionomycin to generate mature model DCs, prior to coculture. Representative histograms showing the proliferation of CD4^+^ and CD8^+^ T cells under the indicated conditions are presented. *C*, percent cell proliferation of CD4^+^ and CD8^+^ T cells. Each column represents the mean ± SEM of three independent experiments. ns, not significant. ∗∗*p* < 0.01 and ∗∗∗∗*p* < 0.0001 as determined by Tukey’s *post hoc* test. CFSE, carboxyfluorescein succinimidyl ester; DC, dendritic cell; LPS, lipopolysaccharide; rhGM-CSF, recombinant human granulocyte macrophage colony-stimulating factor.

To further examine the functional properties of the DC-like cells, induced by loss of integrin α7-mediated signaling in laminin α2-cultured macrophages, the ability of these cells to stimulate T cell proliferation was tested in a coculture system. T cells were labeled with carboxyfluorescein succinimidyl ester (CFSE) before adding them to the coculture system. The fluorescence intensity of CFSE decreases with each cell division. Neutralizing antibodies were chosen for this experiment instead of siRNA to reduce the chances of integrin α7-rebound expression. Neither laminin-221E8 nor the neutralizing antibody alone affected CD4^+^ or CD8^+^ T cell proliferation (Fig. 4, *A* and *C*). When cocultured with the DC-like cells, both CD4^+^ and CD8^+^ T cell proliferation significantly increased compared to coculture with THP-1 macrophages. Furthermore, LPS stimulation caused the DC-like cells to promote CD4^+^ and CD8^+^ T cell proliferation, as indicated by the decrease in CFSE fluorescence (Fig. 4, *A* and *C*). CD4^+^ and CD8^+^ T cells proliferated 1.5 to 2 times more when cocultured with cytokine-treated THP-1 cells (used as model DCs), compared coculture with DC-like cells, under both immature and mature DC conditions (Fig. 4, *B* and *C*).

### Functional inhibition of integrin α7 induces a decrease in PI3K p85α levels and an increase in AKT activation in THP-1 macrophages cultured on laminin α2 chains

**Figure 5:**
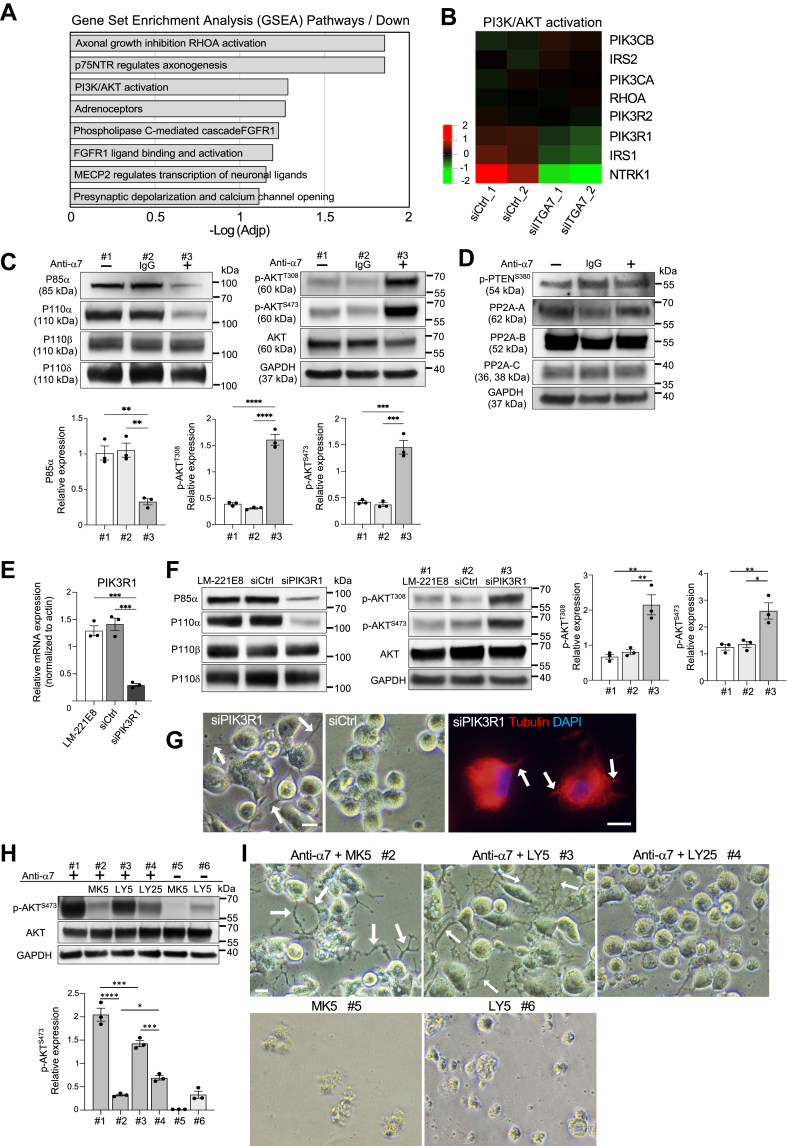
**Functional blocking of integrin α7 induces a decrease in p85α levels and increases AKT activation in THP-1 macrophages on laminin-221E8**. *A*, gene set enrichment analysis (GSEA) of downregulated genes in integrin α7 siRNA-transfected macrophages compared with control. *B*, expression heatmap enriched for signaling by PI3K/AKT activation in RNA-Seq data. Integrin α7-targeted small interfering RNA (siITGA7)-transfected cells compared with nonspecific control siRNA (siCtrl) (n = 2 for each group). *C*, representative western blot image of the regulatory subunit p85α and the catalytic subunits p110α, p110β, and p110δ of class IA PI3K, and AKT phosphorylation (p-AKT) at Thr308 and Ser473, and total AKT on day 5 under indicated conditions. GAPDH was used as a loading control. Levels of phosphorylated proteins were measured by densitometry, normalized to total protein and GAPDH levels. *D*, representative western blot image of PTEN phosphorylation (p-PTEN) at Ser380 and subunits of PP2A: scaffold (*A*), regulatory (*B*), and catalytic (*C*). GAPDH was used as a loading control. *E*, gene expression levels of PIK3R1 in THP-1-derived macrophages treated with PIK3R1 siRNA (siPIK3R1) or nonspecific siRNA (siCtrl) on laminin (LM)-221E8 on day 5. *F*, representative western blot image for class IA PI3K and p-AKT on day 5 under the conditions indicated. GAPDH was used as a loading control. Levels of phosphorylated proteins were measured by densitometry, normalized to total protein and GAPDH levels. *G*, phase contrast images of THP-1 derived macrophages treated with PIK3R1 siRNA or nonspecific siRNA and immunofluorescent staining for microtubule on laminin-221E8 on day 5. *Arrows* represent short projections. *H*, representative western blot image of p-AKT and total AKT under indicated conditions on day 5. AKT inhibitor (MK-2206: MK and 5 μmol/L: MK5) or the PI3K catalytic subunit (p110α/β/δ) inhibitor (LY294002: LY, 5 μmol/L: LY5, and 25 μmol/L: LY25) was added on day 2, and the cells were cultured for 3 days in the presence or absence of integrin α7 function-blocking antibodies. Levels of phosphorylated proteins were measured by densitometry, normalized to total protein and GAPDH levels. *I*, phase contrast images of THP-1 macrophages treated with MK-2206 (MK) or LY294002 (LY) on laminin-221E8 on day 5. *Arrows* represent dendritic processes. PIK3R1 knockdown experiments were repeated three times independently for gene and protein expressions, respectively. The experiments using inhibitors, MK-2206 or LY294002, were repeated three times independently. Anti-α7: anti-integrin α7 antibody. Values are the mean and ± SEM of three independent experiments. ∗*p* < 0.05, ∗∗*p* < 0.01, ∗∗∗*p* < 0.001, and ∗∗∗∗*p* < 0.0001 as determined by Tukey’s *post hoc* test. The *scale bars* represent 10 μm. PP2A, protein phosphatase 2A; PTEN, phosphatase and tensin homologue; RNA-Seq, RNA sequencing.

To further elucidate the mechanism that induces THP-1 macrophage differentiation to DC-like cells, gene set enrichment analysis (GSEA) was performed using RNA-Seq data. The expression of genes involved in PI3K/AKT activation, which is important for DC differentiation (39), was downregulated in macrophages transfected with α7 integrin siRNA (Fig. 5*A*). Among the PI3K/AKT activation-related targets, the expression of the PI3KR1 gene, which encodes the regulatory subunit p85α of class IA PI3Ks, decreased in integrin α7-knockdown macrophages (Fig. 5*B*). Class IA PI3Ks are heterodimeric proteins composed of p110α, p110β, or p110δ catalytic subunits that constitutively interact with the p85α regulatory subunit (40). Western blotting and quantification confirmed that p85α was significantly reduced in macrophages treated with antibody inhibiting integrin α7 function (Fig. 5*C*). The levels of the catalytic subunit of p110α were also decreased by integrin α7 function-blocking antibodies (Fig. 5*C*). Because PI3K is a canonical key regulator of AKT activity, we determined how the observed reduction in p85α levels of PI3K would affect AKT activation. AKT phosphorylation levels at threonine-308 (T308) and serine-473 (S473) were found to be significantly elevated in macrophages treated with integrin α7 function-blocking antibodies compared to controls (Fig. 5*C*). Next, the expression of two negative regulators of AKT was analyzed: phosphatase and tensin homologue (PTEN) deleted on chromosome 10 and protein phosphatase 2A (PP2A). PTEN converts phosphatidylinositol (PI)-3,4,5-triphosphate (PIP3) to PI-4,5-biphosphate (PIP2) and negatively regulates PI3K/AKT signaling (41). Several reports have shown that the downregulation of p85α leads to a decrease in PTEN activity, increasing AKT activation (42, 43). PP2A is a critical serine/threonine phosphatase that directly inactivates AKT (44). Integrin α2β1, a collagen receptor, was shown to promote PP2A activation and dephosphorylate AKT (45). We did not find alterations in phospho-PTEN or PP2A levels in our experimental model (Fig. 5*D*), indicating that AKT activation in this particular context is not due to downregulation of known negative regulatory factors such as PTEN and PP2A.

Next, based on the hypothesis that decreased expression of p85α promotes AKT phosphorylation on laminin α2 chains, the p85α-encoding *PIK3R1*gene was knocked down with siRNA. *PIK3R1* siRNA reduced *PIK3R1* (Fig. 5*E*) and p85α (Fig. 5*F*) levels. Lower levels of p110α, but not of p110β or p110δ, were observed when PIK3R1 was knocked down (Fig. 5*F*). Consistent with our above-described findings (Fig. 5*C*), the reduced levels of p85α were associated with significantly increased levels of AKT phosphorylation at T308 and S473 on laminin-221E8 upon PIK3R1 knockdown (Fig. 5*F*). However, morphological observations showed that only fine, short projections were induced by PIK3R1 knockdown in THP-1 macrophages cultured on laminin-221E8 (Fig. 5*G*). Next, to determine whether AKT activation contributes to dendritic morphology, the AKT inhibitor MK-2206 was used. MK-2206 in combination with integrin α7 function-blocking antibodies significantly decreased AKT phosphorylation (Fig. 5*H*); however, dendritic morphology was observed (Fig. 5*I*). By contrast, the same dose of MK-2206 in the absence of integrin α7 antibody abolished AKT phosphorylation (Fig. 5*H*) and THP-1 macrophages underwent apoptosis (Fig. 5*I*). We next investigated whether the decrease in p85α levels leads to activation of AKT phosphorylation. PI3K catalytic subunits (p110α/β/γ) inhibitor, LY294002, was used in combination with or without integrin α7 function-blocking antibodies to determine the effect of integrin α7 on AKT activation. LY294002 significantly reduced AKT phosphorylation in a dose-dependent manner (Fig. 5*H*), indicating that PI3K activates AKT in the context of decreased p85α levels. Regarding morphology, 25 μM of LY294002 inhibited dendrite formation (Fig. 5*I*), although AKT activation was higher than that with MK-2206 (Fig. 5*H*). Taken together, these results indicate that loss of integrin α7 in THP-1 macrophages cultured with laminin-α2 chains induces a reduction in p85α levels and an increase in AKT activation. Furthermore, whereas PI3K activation is required for morphological changes, AKT phosphorylation is not always a prerequisite and may thus not have a causal role.

### Laminin isoforms affect the kinetics of GM-CSF-induced primary human macrophages

**Figure 6:**
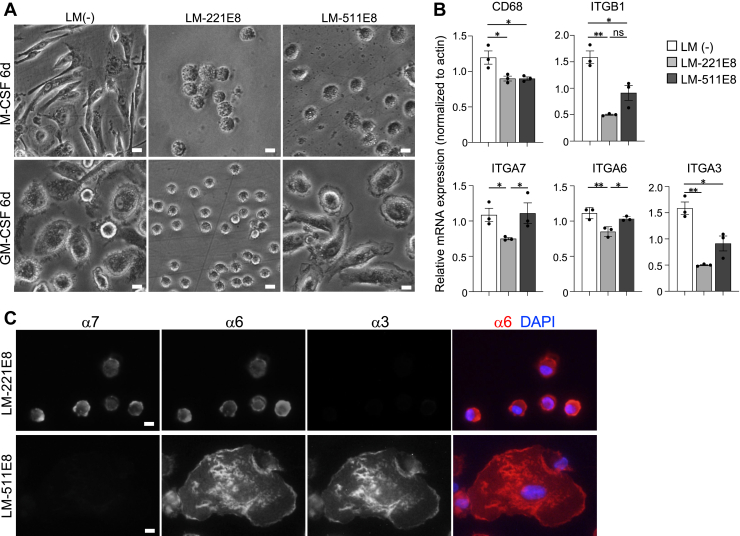
**Laminin isoforms differentially affect GM-CSF-induced human primary macrophage kinetics**. *A*, day 6 morphology of M-CSF or GM-CSF stimulated monocytes cultured on laminin (LM)-221E8/-511E8 or without laminin coat (LM-). M-CSF stimulated monocytes are floating on LM-221E8 or -511E8 coated plates. *B*, gene expression levels of CD68, integrin β1 (ITGB1), integrin α7 (ITGA7), integrin α6 (ITGA6), integrin α3 (ITGA3) in GM-CSF stimulated monocytes cultured on LM-221E8/-511E8 or LM (−). Data are presented as the mean ± SEM of three independent experiments. ∗*p* < 0.05 and ∗∗*p* < 0.01 as determined by Tukey’s *post hoc* test. *C*, multiple immunofluorescent staining for laminin-related integrins (α7, α6, and α3) in GM-CSF-stimulated monocyte-derived macrophages cultured on LM-221E8 or -511E8. Immunostaining was performed four times independently, imaging a total of 300 to 350 cells per antibody. Nuclei were counterstained with DAPI. The *scale bars* represent 10 μm. DAPI, 4′,6-diamidino-2-phenylindole; GM-CSF, granulocyte macrophage colony-stimulating factor.

To confirm the observation in THP-1-derived macrophages with primary human macrophages, two types of primary monocyte-derived macrophages (MDMs) were tested: MDMs differentiated using macrophage colony-stimulating factor (M-CSF) or granulocyte macrophage colony-stimulating factor (GM-CSF) (46). The MDMs stimulated with M-CSF did not adhere to laminin-221E or −511E8 even after 6 days (Fig. 6*A*), but in contrast, did adhere to non-laminin-coated dishes on day 1. The present findings are consistent with the previous report (47) showing that M-CSF-stimulated macrophages did not attach to laminin. Therefore, GM-CSF-derived MDMs were used in subsequent experiments and are referred to as MDMs. MDMs were adherent and spherical on laminin-221E8 on day 6. In contrast, on laminin-511E8, they were expanded in shape as in the absence of laminin coating (Fig. 6*A*). The mRNA expression levels of CD68, a pan-macrophage marker, in MDMs were significantly lowered by laminin coating (Fig. 6*B*). All laminin related integrins were identified in MDMs (Fig. 6*B*). The expression levels of integrins α7, α6, and α3 were all reduced in cells grown on laminin-211E8 (Fig. 6*B*). Notably, the expression level of integrin α7 was significantly downregulated in MDMs grown on laminin-221E8 (Fig. 6*B*), in contrast to THP-1 derived macrophages; integrin α7 gene expression level was significantly higher when cultured on laminin-α2 than when on laminin-α5 or in the absence of laminin coat (4). This suggests that GM-CSF downregulates integrin α7 gene expression on laminin-α2 chains.

Protein expression was evaluated using anti-integrin α7/6/3 antibodies in MDMs cultured on laminin-221E8 or -511E8-coated dishes on day 6 of culture. MDMs cultured on laminin-221E8 showed staining for integrins α7 and α6, but not integrin α3 (Fig. 6*C*). In contrast, the cells cultured on laminin-511E8 had no integrin α7 localization; however, integrins α6 and α3 were localized throughout the cell (Fig. 6*C*). These immunolocalizations are consistent with the results shown for THP-1 macrophages cultured on laminin-211 or laminin-511 (Fig. 1*A*). Taken together, these data suggest that laminin isoforms affect the kinetics of MDMs.

### MDMs cultured on laminin-221E8 are altered to exhibit a DC-like phenotype

**Figure 7:**
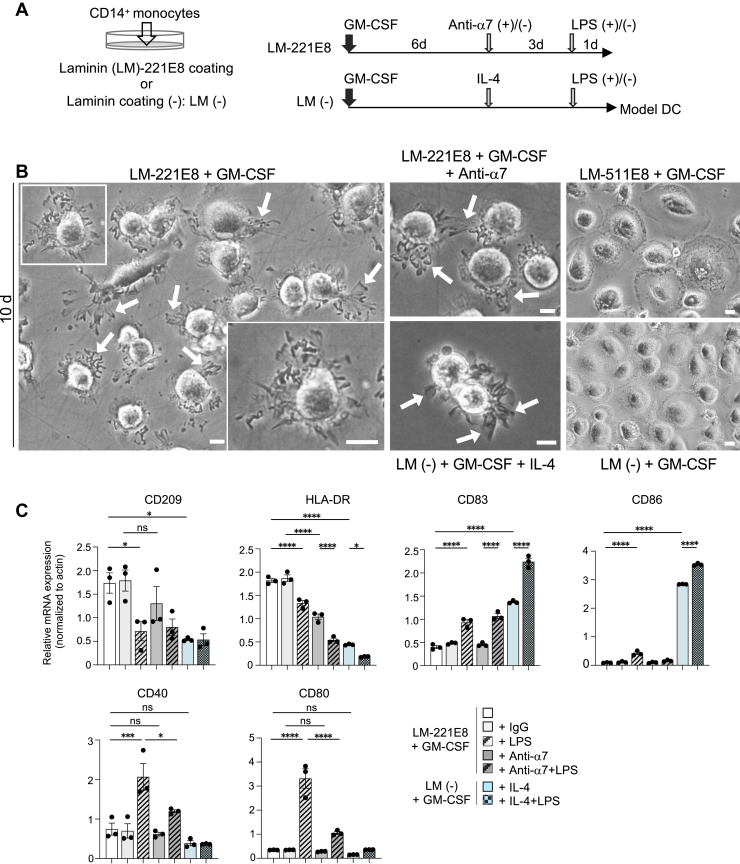
**GM-CSF-stimulated monocyte-derived macrophages cultured on laminin-221E8 are altered to exhibit a DC-like phenotype**. *A*, schematic diagram of the experimental method using human peripheral blood CD14^+^ monocytes. *B*, day 10 morphology of GM-CSF derived macrophages cultured on laminin (LM)-221E8/-511E8 or without laminin coat (LM-). *Arrows* indicate veil-like membrane projections. A closer image of the boxed area is shown in the inset. The scale bars represent 10 μm. *C*, analysis of gene expression in monocyte derived macrophages cultured under the conditions indicated. Data are presented as the mean ± SEM of three independent experiments. ∗*p* < 0.05, ∗∗∗*p* < 0.001, and ∗∗∗∗*p* < 0.0001 as determined by Tukey’s *post hoc* test. Anti-α7: anti-integrin α7 antibody. DC, dendritic cell; GM-CSF, granulocyte macrophage colony-stimulating factor.

Monocytes have been shown to be important DC precursor cells both *in vitro* and *in vivo* (48). Monocyte-derived DCs can be generated by monocyte cultivation with GM-CSF and IL-4 *in vitro* (49). To assess the effect of integrin α7 on laminin α2 chains in MDMs, they were further cultured in the presence or absence of anti-integrin α7 and compared to the known monocyte-derived DC models (Fig. 7*A*). Unexpectedly, MDMs cultured on laminin-221E8 with only GM-CSF added showed dendritic morphology with veil-like membrane projections by day 10 of culture (Fig. 7*B*). The dendritic morphology was also evident in the presence of antibodies inhibiting integrin α7 function. Model DCs cultured with GM-CSF and IL-4 were detached from the plate and showed veil-like membrane projections as previously reported (49). In contrast, MDMs cultured on laminin-511E8 or without the laminin coat showed giant round cells resembling fried eggs (50).

Expression of DC markers CD209, HLA-DR, as well as the LPS-stimulated costimulatory molecules CD40 and CD80, was significantly higher in MDMs cultured on laminin-221E8 compared to known DC models (Fig. 7*C*). CD83, a marker of mature DCs (51), was significantly increased in cells on laminin-221E8 and in DC models upon LPS stimulation. In contrast, the costimulatory molecule CD86 was highly expressed in the DC model. Antibodies inhibiting integrin α7 function reduced HLA-DR expression but had no effect on other factors (Fig. 7*C*). Taken together, these findings suggest that GM-CSF-derived MDMs cultured on laminin α2 chains acquired a DC-like phenotype.

## Discussion

This study shows that the loss of integrin α7 signaling in THP-1-derived macrophages cultured on α2-containing laminin isoforms induces dendrite formation. These cells exhibited active endocytosis, increased expression of DC markers and T cell costimulatory molecules, and could stimulate T cell proliferation, suggesting that these cells acquired a DC-like phenotype.

The dendrites extending from THP-1 macrophages were composed of microtubules and MAP2. Microtubules play important roles in various cellular functions, such as cytokinesis, cellular morphogenesis, and intracellular transport, and their physical properties strongly influence these functions (52). MAPs are a set of proteins that directly bind microtubules and stabilize them (37). MAP2 is predominantly detected in neuronal dendrites, where it not only maintains dendritic structure but also influences intracellular trafficking (38). The ability of DCs to take up antigens is closely related to their endocytic capability. In DCs, FITC-dextran uptake occurs through macropinocytosis—a process by which large amounts of extracellular fluid are taken up in digestive vesicles (32). In this study, numerous puncta of FITC-dextran, similar in size to macropinosomes, were detected in the dendrites and cell bodies, suggesting that FITC-dextran is taken up by macropinocytosis, and microtubules and MAP2 are involved in the active endocytosis of FITC-dextran.

Immunofluorescent localization of laminin-associated integrins α7/α6/α3 was different between laminin α2 and α5 chains in both THP-1-derived macrophages and MDMs. The isoforms of each laminin differ in their binding specificities and affinities for laminin-related integrins (15), which may cause the differences in integrin localization detected in this study. A previous study demonstrated that macrophages cultured on laminin-211 do not show morphological changes when cultured with neutralizing antibodies against integrin α3 or α6 (4). This may be due to the absence of integrin α3 localization on laminin α2 and the reported inside-out regulation of integrin α6 in macrophages (53). By contrast, no induction of dendritic processes was observed on laminin-511 due to blocked integrin α7 function. On laminin-511, macrophages expanded, and integrin α3 was localized throughout the cells. Our previous study indicated that neutralizing antibodies against integrin α3 prevent macrophage expansion and increased mRNA expression of CD163 and IL-10, an inducer of CD163 (54), on laminin-511 (4). Thus, integrin α7 is the only integrin that prevents macrophages cultured on laminin α2 chains from differentiating into DC-like cells. In other words, laminin α2–integrin α7 signaling prevents the differentiation process. Taken together, our present and previous data suggest that laminin-related integrins have distinctive functions on laminin isoforms in macrophages, in addition to their basic function of adhesion to laminins in the extracellular matrix.

The kinase AKT plays a central role in signaling pathways that regulate metabolism and cellular transformation (41). Research on the immunological functions of the AKT signaling network is an active field, and AKT signaling is crucial for DCs, including survival by inhibiting apoptosis, differentiation, and T cell activation (55, 56). PI3K signaling results in the activation of multiple signaling pathways. Mutation or decrease of p85α induced the activation of PI3K and AKT (40, 57, 58). The molecular balance between the regulatory and catalytic subunits of PI3K is critical in maintaining activity. The p85α subunit is more abundant than the p110 subunit, and free p85α performs a key inhibitory function in the PI3K pathway by competing with the p85α-p110 complex for receptor binding (58). We found that inhibition of integrin α7 function decreased p85α levels and increased AKT activation. AKT activation was also caused by the knockdown of PIK3RI in THP-1 macrophages cultured on laminin α2 chains, suggesting that the decrease of free p85α increased AKT activation. The catalytic subunits of class I PI3K isoforms have distinctly different expression profiles and functions—p110α and p110β are ubiquitously expressed, whereas p110δ is predominantly expressed in immune cells (59). Therefore, PI3K signaling plays essential roles in the immune response (60, 61). In DCs, PI3K p110δ is crucial for antigen presentation, cytokine production, and AKT phosphorylation (62). The decrease of p85α levels was associated with decreased p110α levels, but the level of p110δ was unchanged, suggesting that this balance could lead to the increased binding of p85α to p110δ to regulate DC phenotype. However, the detailed signaling events are not completely understood. Future studies are required to determine the mechanism by which the loss of integrin α7 signaling in macrophages cultured on the laminin α2 chain reduces the levels of the p85α subunit and how the balance between p110α, -β, and -δ levels is regulated.

The hematopoietic growth factors GM-CSF and M-CSF regulate the proliferation and differentiation of macrophages from monocytes (50). Macrophages induced by GM-CSF and M-CSF differ in shape and cell surface antigens as well as expression of integrin molecules (47). CSFs affect integrin expression, as αVβ5 is expressed in M-CSF-induced macrophages and αVβ3 in GM-CSF-induced macrophages. In the present study, macrophages induced by M-CSF did not adhere to laminin isoforms, which is consistent with the previous report (47). On the other hand, GM-CSF-induced macrophages adhered to both laminin-α2 and α5 chains, but their integrin expression was significantly reduced on laminin α2, especially integrin α7 (Fig. 6*B*). Culturing THP-1 macrophages on laminin-α2 chains significantly increased integrin α7 expression (4), and lowering the integrin α7 induced DC-like cells, suggesting that integrin α7, reduced by GM-CSF, induced MDMs to become DC-like cells. *In vivo*, GM-CSF is prominently produced by T cells in inflamed tissue and is also produced by tumor cells (63); further studies are needed to determine how GM-CSF affects the environment in which macrophages associate with laminin-α2 chains.

In clinical studies, integrin α7 functioned as a biomarker and a therapeutic target in several types of tumors (19, 20, 21). In glioblastoma, the most lethal primary brain tumor, laminin α2 is aberrantly expressed (64), and antibody-based inhibition of integrin α7 signaling was effective *in vitro* and *in vivo* (21). Integrin α7 has been suggested to be critical in other tumors, and blocking integrin α7 is expected to be clinically useful (22, 23). In addition, tumor-associated macrophages and microglia, which are a highly heterogeneous and abundant population of immune cells in tumor mass, have also been suggested to be potential therapeutic targets (65, 66). In recent years, DCs have attracted attention due to their potential for various clinical applications, such as immune cell therapy (24). This study suggests that DCs are not only regulated through signal transduction from various cytokine environments but also by laminin α2-and integrin α7-mediated cell adhesion in macrophages, which is a novel finding. Thus, understanding these detailed signals will be important for future therapeutic strategies that target integrin α7.

## Experimental procedures

### THP-1 cell line and cell culture

Monocytic cell line THP-l was maintained in Dulbecco's modified Eagle's medium (DMEM) (Wako, 041–29775) supplemented with 10% fetal bovine serum (Sigma-Aldrich), 10 IU/ml penicillin, and 10 μg/ml streptomycin (Wako) at 37 °C with 5% CO_2_. In addition, 24-well tissue culture plates (Corning) were coated with laminin-211, laminin-221, or laminin511 (BioLamina) at 1.25 μg/cm^2^ in PBS. Coating with recombinant laminin E8 fragments of α2 (LM-221E8, iMatrix, Matrixome, Inc) was performed at a density of 1.25 μg/cm^2^. To induce the differentiation of THP-1 cells to a macrophage phenotype, the cells were seeded at 0.2 × 10^6^ cells/well with culture media containing 5 ng/ml phorbol 12-myristate 13-acetate (PMA) (Sigma-Aldrich), as described (4). After 2 days, THP-1 macrophages were washed and incubated in culture media alone for 3 days.

### Antibodies, reagents, and fluorescent dyes

All the antibodies for this study are provided in Table S1. Neutralizing antibodies specific for integrin α7 (DSHB Hybridoma Product 9.1 ITGA7, deposited by Kramer R. H.) were added to the culture medium at 10 μg/ml from the start to the end of the experiment. An isotype control IgG was obtained from Bio X Cell (BE0366). LY294002 and MK-2206 were obtained from Selleck. Phalloidin-iFluor 488 (AAT Bioquest) was used to label F-actin. Recombinant human (rh) IL-4, rhM-CSF, and rhGM-CSF were purchased from R&D Systems. rhTNF-α was purchased from Gibco and LPS and ionomycin from Sigma-Aldrich.

### Generation of DCs from THP-1 cells using cytokines

THP-1 cells were induced into immature and mature DCs using cytokines as described (29). To generate immature DCs, THP-1 cells were cultured in a culture medium containing 10% fetal bovine serum, rhIL-4 (100 ng/ml), and rhGM-CSF (100 ng/ml) for 5 days. To generate mature DCs, immature DCs were suspended in serum-free culture medium containing rhIL-4 (200 ng/ml), rhGM-CSF (100 ng/ml), rhTNF-α (20 ng/ml), and ionomycin (200 ng/ml), and cultured for 1 day.

### Generation of human primary monocyte-derived macrophages and DCs

Human peripheral blood CD14^+^ monocytes were obtained from Veritas. These monocytic cells were cultured for 6 days in the DMEM (Wako, 041–29775) supplemented with 10% fetal bovine serum (Sigma-Aldrich), 10 IU/ml penicillin, and 10 μg/ml streptomycin (Wako), in the presence of rhM-CSF (50 ng/ml) or rhGM-CSF (50 ng/ml) to get macrophages as previously reported (46). Once differentiated, the macrophages were further cultured for 4 days with rhGM-CSF (50 ng/ml) and IL-4 (30 ng/ml) for generation DCs, as previously described (46). Mature DCs were obtained by the addition of LPS (1 μg/ml) at the last 1 day of culture (67).

### Gene silencing

siRNAs were obtained from QIAGEN: FlexiTube GeneSolution GS3679 for ITGA7. siRNA Name: Hs_ITGA7_10, Cat# SI05137398, Target sequence: ATGGATGTGGATGGAACAACA; Hs_ITGA7_9, Cat# SI05137391, Target sequence: CTGGATGTGGACAGTAGGGAT; Hs_ITGA7_1, Cat# SI00034223, Target sequence: CCCAGTGATGGTATACTTGGA; FlexiTube GeneSolution GS5295 for PIK3R1, siRNA Name: Hs_PIK3R1_10, Cat# SI04379417, Target sequence: CACTACCGGAATGAATCTCTA; a nontarget control siRNA Cat# 1027281. siRNA transfection was performed with Lipofectamine RNAiMAX (Thermo Fisher Scientific) according to the manufacturer’s protocol. Two days post transfection of siRNA with PMA, the medium was replaced with fresh complete DMEM, and the cells were maintained for 3 days. The efficiency of knockdown was verified by quantitative real-time PCR and western blotting.

### Immunofluorescent staining

THP-1 cells were plated on laminin-211, 221E8, or laminin-511-coated glass-bottom dishes (Iwaki), activated with PMA for 2 days, washed and incubated in culture media for 3 days in the absence or presence of the function blocking antibodies against integrin α7, and fixed with 4% paraformaldehyde for 20 min. Immunostaining was performed using the primary antibodies, and the appropriate secondary antibodies. The samples were counterstained with ProLong Diamond Antifade Mountant with 4′,6-diamidino-2-phenylindole (DAPI) (Thermo Fisher Scientific, P36962). All fluorescent images were obtained using a Nikon E−800 fluorescent microscope equipped with a digital microscope camera (DP-80, Olympus).

### Quantitative real-time PCR

Total RNA was extracted from cells using TRIzol Reagent (Life Technologies) in accordance with the manufacturer’s instructions. The extracted RNA was quantified using a NanoDrop microvolume spectrophotometer (Thermo Fisher Scientific). PrimeScript RT Master Mix (Takara Bio) was used to synthesize complementary DNA. Quantitative real-time PCR was performed using QuantStudio 3 Real-Time PCR System (Thermo Fisher Scientific) with gene-specific primers and TB Green Premix Ex Taq II (Takara Bio), in accordance with the manufacturer’s instructions. Data were analyzed using the delta-delta Ct method with expression normalized to the housekeeping gene β-actin. Each experiment was performed three times. Primer sequences are listed in Table S2.

### Western blotting

Cells were washed with Hank’s balanced salt solution and lysed in cell lysis buffer (20 mM Tris–HCl [pH 7.5], 150 mM NaCl, 1 mM Na_2_EDTA, 1 mM EGTA, 1% Triton, 2.5 mM sodium pyrophosphate, 1 mM beta-glycerophosphate, 1 mM Na_3_VO_4_, and 1 μg/ml leupeptin; Cell Signaling Technology, 9803) supplemented with protease inhibitor (Cell Signaling Technology, 8553) and phosphatase inhibitor (Nacalai Tesque, 07574). The extracted proteins were mixed with LDS Sample Buffer (Thermo Fisher Scientific, NP0007), heated at 70 °C for 10 min, loaded onto gels (Thermo Fisher Scientific, NW04120BOX), and transferred onto a polyvinylidene difluoride membrane (Thermo Fisher Scientific, LC2002). After transferring, the membrane was blocked and incubated overnight at 4 °C with primary antibodies. This was followed by an incubation with horseradish peroxidase-conjugated secondary antibodies for 1 h at room temperature. Chemiluminescence was induced by incubating the membrane for 3 min in the Western Blotting Detection Reagent (Cytiva, RPN2235) and detected using an ImageQuant LAS-4000 mini (Cytiva). Western blotting was performed in triplicate. Densitometry was performed using the ImageJ program. To quantify levels of protein phosphorylation, the optical density of bands for phosphorylation was normalized to total protein and GAPDH.

### Endocytosis assay

The endocytic capacity of THP-1 cells treated with integrin α7-neutralizing antibodies was tested by assaying the uptake of FITC-dextran (TdB Labs). The endocytic tracer was added to a final concentration of 1 mg/ml for 30 min at 37 °C with 5% CO_2_. Cells incubated with FITC-dextran on ice were used as background controls. The cells were washed with ice-cold Flow Cytometry Staining Buffer (Funakoshi). The uptake of FITC-dextran was quantified by flow cytometry (NovoCyte Flow Cytometer; ACEA Biosciences). To morphologically analyze endocytosis, cells were cultured on an 8-well chamber slide glass (Matsunami) incubated with FITC-dextran (1 mg/ml) at 37 °C or on ice for 30 min. Cells were washed with ice-cold PBS containing 1% fetal calf serum and 0.1% NaN_3_, fixed in 4% paraformaldehyde for 10 min, washed, covered by a coverslip with antifade mountant (Invitrogen), and examined under a Nikon E−800 fluorescent microscope equipped with a digital microscope camera (DP-80, Olympus).

### T cell proliferation assay

CD4^+^ and CD8^+^ T cells were obtained from Veritas. For the stimulation and proliferation assays, T cells were stained with 5 μM CFSE using CellTrace CFSE Cell Proliferation Kit (Thermo Fisher Scientific, C34554). Briefly, T cells were incubated in CFSE solution at 37 °C for 20 min. The cells were then washed briefly in complete medium. Triplicates of stimulator cells (2 × 10^5^) and CFSE-stained T cells (6 × 10^5^) were cocultured in laminin-221E8-coated 24-well plates in the presence of integrin α7 function-blocking antibodies. LPS (1 μg/ml) was used to activate stimulator cells for 1 day before coculture. As controls, the T cells were cocultured with THP-1 macrophages without anti-integrin α7 or with THP-1 cells treated with cytokines (model DCs). The cells were cocultured for 6 days and collected by heavy pipetting and washed three times with Flow Cytometry Staining Buffer (R&D System, FC001). Fluorescence was measured using flow cytometry.

### RNA sequencing

A next-generation sequencing library was prepared using Illumina Stranded mRNA Prep, Ligation (Illumina). Libraries with different indices were multiplexed and loaded onto a NovaSeq 6000 instrument (Illumina) with a 2 × 150-bp paired-end configuration. The sequences were processed and analyzed by Genome-Lead Inc.

### Enrichment and pathway analysis of differentially expressed genes

Differentially expressed genes were identified based on comparisons between integrin α7 siRNA and control siRNA groups using DESeq2. Genes with an empirical false discovery rate of <0.1 and a fold-change of >2.0 were considered differentially expressed genes. Enrichment analysis of differentially expressed genes was performed using the Gene Ontology molecular function and Kyoto Encyclopedia of Genes and Genomes databases. GSEA was conducted using the fgsea package. A false discovery rate of <0.10 was considered statistically significant for GSEA. Bioinformatic analyses were conducted using iDEP 1.0 (Ge *et al*. BMC Bioinformatics 19, 534. 2018).

### Statistical analysis

Quantitative real-time PCR and cell fluorescence data obtained by flow cytometry were evaluated using one-way ANOVA with Tukey’s *post hoc* test or an unpaired *t* test. All statistical analyses were conducted using GraphPad Prism 9 (GraphPad Software, Inc).

## Data availability

All data necessary to evaluate the conclusions of this study are contained within the article or supporting information.

## Supporting information

This article contains supporting information.

## Conflict of interest

The authors declare that they have no conflict of interests with the contents of this article.
