# Supplementary material for: Loss of integrin alpha7-mediated signaling induces a dendritic cell-like phenotype in macrophages cultured on laminin-211/221 isoforms
Source: J Biol Chem. 2025 Jun 25;301(8):110419. doi: 10.1016/j.jbc.2025.110419 (PMC12301786; doi:10.1016/j.jbc.2025.110419)
Supplement: Supplementary Material [file mmc1.docx]

**Loss of integrin alpha7-mediated signaling induces a dendritic cell-like phenotype in macrophages cultured on** **laminin-211/221 isoforms**

Nagako Yoshiba, Tomoki Maekawa, Kiyotoshi Sekiguchi, et al.

Table S1. Antibodies used in the present study

| Target | Host | Dilution (Experiment) | Clone | Manufacturer | Cat # |
| --- | --- | --- | --- | --- | --- |
| Integrin α7 | Mouse | 1:100 (WB), 1:20 (IF) | 9.1 ITGA7 | DSHB Hybridoma | 2721955 |
| Integrin α7 | Rabbit | 1:100 (IF) | Polyclonal | Bioss | bs-1816R |
| Integrin α6 | Rat | 1:100 (IF) | GoH3 | R&D Systems | MAB13501 |
| Integrin α3 | Mouse | 1:200 (IF) | ASC-1 | Sigma-Aldrich | MAB2056 |
| Tubulin | Rat | 1:300 (IF) | YOL1/34 | abcam | ab6161 |
| MAP2 | Rabbit | 1:200 (IF) | Polyclonal | proteintech | 17490-1 |
| PI3K p85α | Rabbit | 1:1000 (WB) | 19H8 | Cell Signaling | 4257 |
| PI3K p110α | Rabbit | 1:1000 (WB) | C73F8 | Cell Signaling | 4249 |
| PI3K p110β | Rabbit | 1:1000 (WB) | C33D4 | Cell Signaling | 3011 |
| PI3K p110δ | Rabbit | 1:1000 (WB) | D1Q7R | Cell Signaling | 34050 |
| Phospho-AKT^T308^ | Rabbit | 1:2000 (WB) | D25E6 | Cell Signaling | 13038 |
| Phospho-AKT^S473^ | Rabbit | 1:2000 (WB) | D9E | Cell Signaling | 4060 |
| AKT | Rabbit | 1:1000 (WB) | Polyclonal | Cell Signaling | 9272 |
| Phospho-PTEN^S380^ | Rabbit | 1:1000 (WB) | Polyclonal | Cell Signaling | 9551 |
| PP2A-A | Rabbit | 1:1000 (WB) | 81G5 | Cell Signaling | 9780 |
| PP2A-B | Rabbit | 1:1000 (WB) | 100C1 | Cell Signaling | 9780 |
| PP2A-C | Rabbit | 1:1000 (WB) | 52F8 | Cell Signaling | 9780 |
| GAPDH | Rabbit | 1:5000 (WB) | D16H11 | Cell Signaling | 8884 |
| β-actin | Rabbit | 1:5000 (WB) | 13E5 | Cell Signaling | 5125 |
| Anti-rabbit HRP | Swine | 1:3000 (WB) |  | DAKO | P 0399 |
| Anti-rabbit Alexa 488 | Goat | 1:200 (IF) |  | Invitrogen | A11034 |
| Anti-rabbit Alexa 546 | Goat | 1:200 (IF) |  | Invitrogen | A11010 |
| Anti-mouse Alexa 647 | Goat | 1:200 (IF) |  | Invitrogen | A21237 |
| Anti-rat Cy3 | Goat | 1:800 (IF) |  | Jackson | 112165167 |
|  |  |  |  |  |  |
|  |  |  |  |  |  |
|  |  |  |  |  |  |

Table S2. Primer sequences for quantitative RT-PCR analysis

| Integrin α7 | Forward: GCTGCCCACTCTACAGCTTTGAC |
| --- | --- |
|  | Reverse: ACAATCACTTCCAGGGACTTCACA |
| Integrin α6  Integrin α3  Integrin β1  CD68  CD209 | Forward: GGACAGCAAGGCGTCTCTTATT  Reverse: CGGCAGCAGCAGTCACATCAA  Forward: TCAACCTGGATACCCGATTCC  Reverse: GCTCTGTCTGCCGATGGAG  Forward: CCGCGCGGAAAAGATGAAT  Reverse: CCACAATTTGGCCCTGCTTG  Forward: GCTACATGGCGGTGGAGTACAA  Reverse: ATGATGAGAGGCAGCAAGATGG  Forward: CCAAGCAGCAGCAAATCTATCAAG |
|  | Reverse: GCTTCTCCTGGATGGGACTTTC |
| CD83 | Forward: TTATTGGAGGGTGGTGAAGAGAGGATG |
|  | Reverse: TGTCACTCTCAAGATCACCTTGCC |
| HLA-DR | Forward: GGACAAAGCCAACCTGGAAA |
|  | Reverse: AGGACGTTGGGCTCTCTCAG |
| CD40 | Forward: CCTCGCCATGGTTCGTCTGCC |
|  | Reverse: AGCCAGGAAGATCGTCGGGA |
| CD80 | Forward: CATCTGACGAGGGCACATAC |
|  | Reverse: GGTGTAGGGAAGTCAGCT |
| CD86 | Forward: CAACGGAATTAGGAAGAC |
|  | Reverse: CTCTGTATGCAAGTTTCC |
| MAP2 | Forward: TCAGAGGCAATGACCTTACC |
|  | Reverse: GTGGTAGGCTCTTGGTCTTT |
| PIK3R1 | Forward: CGCCTCTTCTTATCAAGCTCGTG |
|  | Reverse: GAAGCTGTCGTAATTCTGCCAGG |
| β-actin | Forward: GATTCCTATGTGGGCGACGA |
|  | Reverse: GTGGTGCCAGATTTTCTCCA |
